# School-based WASH facilities and menstrual hygiene management: a systematic review of impacts on adolescent girls in South Asia, Sub-Saharan Africa, and Southeast Asia

**DOI:** 10.1186/s12905-026-04796-2

**Published:** 2026-08-17

**Authors:** Sparsha Jain, Manjushree G. Naik

**Affiliations:** Manipal Institute of Communication, Manipal Academy of Higher Education (MAHE), Karnataka, India

**Keywords:** Menstrual hygiene management, Adolescent girls, School WASH, Social ecological model, Low- and middle-income countries

## Abstract

**Background:**

Menstrual health and hygiene are crucial for achieving Sustainable Development Goals, such as SDG 3 (Good Health and Well-being), SDG 4 (Quality Education), SDG 5 (Gender Equality), and SDG 6 (Clean Water and Sanitation), yet they remain underprioritized in global health and education agendas. Millions of school-going girls, particularly in low- and middle-income countries, continue to lack access to accurate information, affordable products, safe facilities, and stigma-free environments for managing menstruation with dignity. The inequities are rooted in structural, cultural, and institutional barriers and underscore the need for multilevel, system-oriented approaches to menstrual health.

**Methods:**

This systematic review aimed to synthesise evidence on how school-based Water, Sanitation, and Hygiene (WASH) facilities influence menstrual hygiene management among adolescent schoolgirls in South Asia, Sub-Saharan Africa, and Southeast Asia. Studies were identified through PubMed, Scopus, Web of Science, JSTOR, and grey literature searches. Eligible primary studies focused on adolescent schoolgirls (10–19 years) and school-based Water, Sanitation, and Hygiene (WASH) environments. Findings were thematically synthesised across five Social Ecological Model (SEM) levels: individual, interpersonal, organisational, community, and policy.

**Results:**

Across settings, menstrual health was shaped by a complex interplay of cognitive, social, infrastructural, and policy determinants. At the individual level, awareness has improved, yet biological understanding remains limited by affordability and stigma. Interpersonal dynamics were dominated by mothers and constrained by silence among fathers, and how teachers mediate girls’ comfort and coping. Organisational barriers, reported inadequate WASH, privacy, and disposal facilities, form the primary bottleneck linking knowledge to behaviour. Community norms of pollution, shame, and restriction reinforce secrecy and absenteeism, while policy frameworks remain fragmented, underfunded, and poorly monitored, with weak integration of Menstrual Hygiene Management (MHM) into education systems. Cross-level analysis revealed that knowledge gains fail to translate into practice without enabling infrastructure, supportive social networks, and institutional accountability.

**Conclusion:**

Menstrual health inequities among adolescent girls are ecological rather than individual. Sustainable progress requires rights-based action across SEM levels: inclusive policy and financing; gender-sensitive, well-maintained school WASH; trained and empathetic educators; community norm transformation; and psychosocial well-being integrated into monitoring frameworks. Only through such system-wide alignment can menstrual health become a public guarantee of dignity, equity, and educational opportunity.

**Supplementary Information:**

The online version contains supplementary material available at https://doi.org/10.1186/s12905-026-04796-2.

## Introduction

Menstrual health and hygiene are fundamental human rights and an essential component of global well-being. They are closely linked to the realisation of several Sustainable Development Goals (SDGs), including SDG 3 (Good Health and Well-being), SDG 4 (Quality Education), SDG 5 (Gender Equality), and SDG 6 (Clean Water and Sanitation). Despite this recognition, menstrual health continues to be a neglected area within public health and education agendas. Many individuals, especially adolescent girls in low- and middle-income countries (LMICs), lack access to accurate information, affordable products, safe sanitation facilities, and supportive social environments necessary for managing menstruation with dignity [1]. These barriers are compounded by deep-rooted gender norms, unequal education systems, and limited sanitation infrastructure, collectively sustaining menstrual inequities.

The UNICEF–WHO report Progress on Drinking Water, Sanitation, and Hygiene in Schools 2015–2023: Special Focus on Menstrual Health underscores the magnitude of these disparities [1]. Globally, only two in five schools (39%) provide menstrual health education, with substantial variation across regions, 84% of secondary schools in Central and Southern Asia compared with just 34% of primary schools. Fewer than one in three schools (31%) worldwide have bins for menstrual waste disposal in girls’ toilets, dropping to 17% in Least Developed Countries and 11% in sub-Saharan Africa. Access to menstrual products is even lower, with only 12% of schools in sub-Saharan Africa offering them for free or at a low cost. Moreover, the absence of private toilets with clean water and soap compromises students’ ability to manage menstruation hygienically [1–3]. These deficiencies reinforce stigma, absenteeism, and loss of dignity for millions of schoolgirls.

In Ethiopia, fewer than half of the surveyed girls had prior knowledge of menstruation before menarche, and many experienced fear and embarrassment when it began [1–3]. Teacher preparedness also remains limited; there are no global datasets tracking educators trained to deliver menstrual health education. Only thirty countries, one-third of which are in sub-Saharan Africa, monitor even one recommended menstrual health indicator, illustrating gaps in accountability and policy monitoring. While some nations, such as Zambia and the Philippines, have made significant strides, global evidence underscores the pressing need for coordinated action through enhanced education, improved infrastructure, and increased financing.

These menstrual health inequities are nested within a broader crisis in Water, Sanitation, and Hygiene (WASH) services. One in five schoolchildren globally (447 million) still lack access to basic drinking water, a similar number (427 million) lack sanitation facilities, and one in three (646 million) have no access to hygiene amenities. Achieving SDG 2030 targets will require at least a twofold acceleration in progress on water and sanitation and a fourfold increase in hygiene improvements [1–3].

Adolescence represents a pivotal stage of physical, emotional, and social development. Around one-quarter of the world’s population is aged 10–24 years, with 90% residing in LMICs and nearly 500 million adolescent girls aged 10–19 living in less-developed regions [4]. In such contexts, the convergence of puberty, gender inequality, and limited sanitation infrastructure exacerbates menstrual health challenges. Although education is strongly associated with improved health and economic outcomes [5–9], menstruation-related barriers often disrupt schooling and undermine this potential. Evidence from LMICs reveals that menstruating schoolgirls frequently face inadequate sanitation facilities, unreliable water supplies, and a lack of privacy [10–16]. The absence of affordable and hygienic menstrual products compels many to rely on unsafe materials, increasing the risk of urogenital infections [17]. In some settings, lack of access to menstrual products has been linked to transactional sex for supplies [18, 19]. Combined with restrictive social taboos and insufficient institutional support, these conditions perpetuate shame, confusion, and absenteeism [11, 20, 21].

According to widely used global monitoring language on menstrual hygiene management (MHM), adequate MHM involves using clean materials to absorb menstrual blood, changing them in privacy, washing with soap and water as needed, and having access to facilities for safe disposal [22]. In practice, these conditions remain unmet across much of the developing world. Studies across LMICs document the use of unhygienic absorbents and inadequate washing or drying of reusable materials [23–28]. For example, in Tanzania, only 18% of women reported using sanitary pads [23]; in Nigeria, 31–56% of schoolgirls used cloth or tissue [24, 25]; in Gambia, one-third used sanitary pads [26]; and in India, 43–88% reused cotton cloths [27, 28].

Beyond hygiene, menstruation carries social and cultural meanings that often impose restrictions and isolation [10, 13, 29–33]. In South India, nearly two-thirds of girls described menarche as “shocking or fearful,” usually followed by several days of seclusion [28]. These experiences reinforce stigma and restrict mobility, education, and participation in daily life. International organisations such as WaterAid, Plan International, and the Water Research Commission have documented how inadequate MHM contributes to absenteeism and school dropout [30, 34]. Physical constraints, including the absence of private toilets, soap, or disposal systems, intersect with psychosocial challenges such as fear of leakage, teasing, and ridicule [33–35]. Menstrual pain and dysmenorrhea further exacerbate attendance and concentration difficulties [30, 34].

Health literacy, the capacity to access, process, and use health information, is central to informed menstrual self-care [36, 37]. However, menstrual health literacy remains alarmingly low among adolescents, especially in LMICs [37]. Despite its cross-sectoral relevance, menstrual health has remained marginal in global policy discourse [38–41]. Within the WASH sector, menstrual hygiene management is often subsumed under general sanitation goals, limiting targeted interventions [14, 41]. Global monitoring frameworks continue to emphasise water and sanitation coverage, while hygiene and menstrual health, in particular, remain poorly tracked and underfunded [22, 42]. As The Lancet observed, “The needs of the 300 million women and girls menstruating on any given day remain buried low on the global health agenda” [43].

While previous systematic reviews have established a foundational understanding of MHM, important structural and geographic gaps remain in the literature. For example, Kuhlmann et al. [44] provided a broad overview of MHM in resource-poor countries, focusing primarily on knowledge, attitudes, practices, and cultural taboos. Similarly, the recent review by Head et al. [45] examined the effectiveness of menstrual health interventions in the East Asia and Pacific region and highlighted limitations in theoretical grounding and the measurement of core menstrual health outcomes. However, existing reviews have paid comparatively limited attention to how school-based WASH environments interact with educational participation and menstrual management across diverse low-resource settings. This review addresses these gaps through a targeted tri-regional synthesis focused on South Asia, Sub-Saharan Africa, and Southeast Asia. In addition, unlike previous reviews, this study applies the Social-Ecological Model (SEM) to examine how multilevel determinants interact with school-based WASH environments, including institutional bottlenecks such as inadequate privacy, lack of point-of-use water, and absence of lockable sanitation facilities, which hinder girls’ ability to translate knowledge into safe and dignified menstrual hygiene practices.

The Social-Ecological Model (SEM) provides a useful framework for examining how menstrual health outcomes are shaped by interactions across the individual, interpersonal, institutional, community, and policy levels [46]. By situating menstrual experiences within broader social and structural contexts, SEM facilitates a more comprehensive understanding of the barriers and enabling factors that influence menstrual hygiene management. Accordingly, this review applies the SEM framework to synthesise evidence derived from the included primary studies on how school-based WASH environments affect menstrual hygiene management among adolescent girls in low-resource settings.

## Methods

### Eligibility criteria

This systematic review was conducted to synthesise cross-country evidence from South Asia, Sub-Saharan Africa, and Southeast Asia on how school-based WASH facilities influence menstrual hygiene management (MHM) practices and girls’ educational outcomes, particularly attendance and participation. The review included materials published between 2009 and 2025. The systematic review followed the guidelines outlined in the Preferred Reporting Items for Systematic Reviews and Meta-Analyses (PRISMA), and the protocol for this review was registered in the International Prospective Register of Systematic Reviews - PROSPERO: CRD420251148314.

To ensure a primary evidence synthesis, this review was restricted exclusively to original empirical studies meeting the inclusion criteria. Prior systematic reviews and meta-analyses were excluded from the primary analytical synthesis and were instead drawn upon in the Introduction and Discussion sections to contextualise and support the synthesised primary findings. Studies were included if they focused on adolescent schoolgirls (10–19 years) in South Asia, Sub-Saharan Africa, and Southeast Asia, and school-based WASH facilities influenced menstrual hygiene management (MHM) practices. Studies were excluded if they were conducted outside the regions of interest (South Asia, Sub-Saharan Africa, Southeast Asia) and focused on general WASH outcomes without specific reference to MHM. Papers exclusively addressing adult women, workplaces, or healthcare settings, rather than schools, were also excluded. Only articles published in English were included.

### Information sources and search strategy

A literature search was conducted across multiple databases and sources, including PubMed, Scopus, Web of Science and JSTOR. Additionally, searches were conducted on government portals and Google Scholar to include grey literature. The literature search was completed and last updated in the first week of October 2025.

The search strategy employed a combination of keywords related to menstrual hygiene management, education, and WASH infrastructure, including “menstrual hygiene management”, “girls’ education”, “menstruation”, “school attendance”, “LMIC WASH infrastructure”, “policy implementation”, “menstrual health”, “WASH”, and “school sanitation”, along with geographic identifiers such as India, Sub-Saharan Africa, Southeast Asia, and country names within these regions.

Search syntax was adapted according to the indexing structure and functionality of each database. Searches were limited to English-language publications published between January 2009 and October 2025. Reference lists of included studies and relevant review articles were also manually screened to identify additional eligible studies. The complete database-specific search strategies are provided in Supplementary Material 1.

### Selection and data collection process

The selection process involved screening titles and abstracts for relevance, followed by the removal of duplicates. Study selection and record management were performed using Microsoft Excel. Articles that passed the initial screening underwent a full-text review. The initial screening and selection process was conducted by the primary author (SJ), and an independent validation step of all retrieved records was undertaken by the second author (MN) to resolve any discrepancies and ensure full consistency with the eligibility criteria. Due to variations in the study designs of the papers included in this review, the extracted data included: author(s), journal, study location, study design, setting, target population, intervention, and results.

### Study risk of bias assessment

To evaluate the risk of bias across the included primary literature, methodological quality was appraised using the Mixed Methods Appraisal Tool (MMAT) [47]. The MMAT was selected because it enables a structured quality assessment across diverse primary research designs, including qualitative, non-randomised quantitative, quantitative descriptive, and mixed-methods studies [47]. This tool allowed for a systematic evaluation of methodological quality and potential risk of bias across all included primary studies.

### Synthesis methods

Findings were synthesised narratively and thematically using the Social Ecological Model (SEM) as the guiding analytical framework. Evidence from the included studies was organised across five interrelated levels of influence. At the individual level, themes captured girls’ menstrual knowledge, attitudes, and hygiene practices. The interpersonal level reflected the influence of peers, teachers, and parents in shaping support, communication, and perceptions of stigma surrounding menstruation. The institutional level encompassed the availability and quality of school WASH infrastructure, including access to water, privacy, and facilities for managing menstrual waste. The community level addressed broader socio-cultural factors such as taboos, social norms, and community engagement that affect menstrual experiences. Finally, the policy level examined the presence and implementation of national or regional WASH and MHM frameworks within education systems. This multi-level synthesis provided a comprehensive understanding of the intersecting determinants that influence menstrual hygiene management among schoolgirls in low-resource settings, highlighting how behavioural, environmental, and structural factors collectively shape menstrual health outcomes.

### Ethics

Ethical approval was not required for this review, as it poses minimal risks and utilises previously published and publicly available data.

## Results

### Study selection

**Fig. 1:**
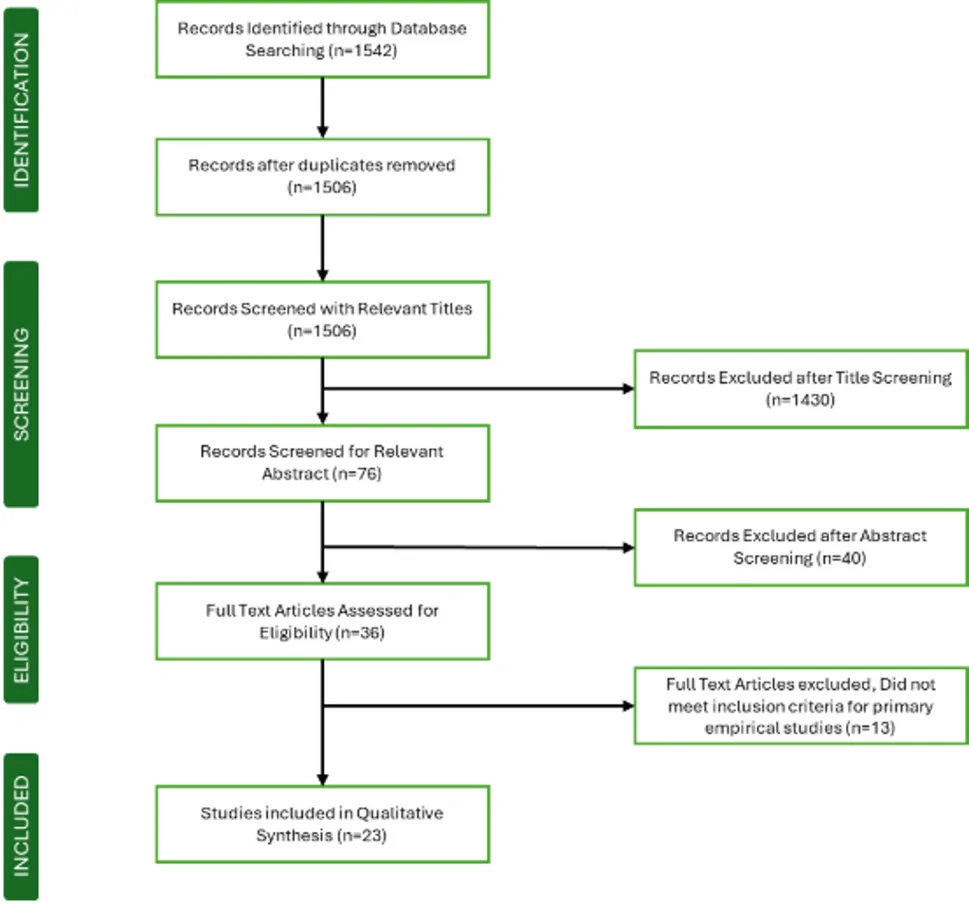
PRISMA flow diagram of the study selection process

A total of 1542 records were identified through database searches. After removing 36 duplicates, 1506 records remained for initial screening. Titles and abstracts were reviewed, resulting in the exclusion of studies that did not focus on adolescent schoolgirls, menstrual health management and WASH interventions in school settings. Following full-text assessment of the 36 retrieved articles, 23 primary studies met the eligibility criteria and were included in the thematic synthesis. The remaining 13 articles, comprising prior systematic reviews, meta-analyses, and broad policy syntheses, were set aside from the primary extraction pool and used instead in the Introduction and Discussion to ground and contextualise the empirical findings. The included studies aim to understand how school-based WASH facilities shape menstrual hygiene management and girls’ education across South Asia, Sub-Saharan Africa, and Southeast Asia, while highlighting policy, infrastructural, and socio-cultural influences (Fig. 1).

### Study characteristics

**Fig. 2:**
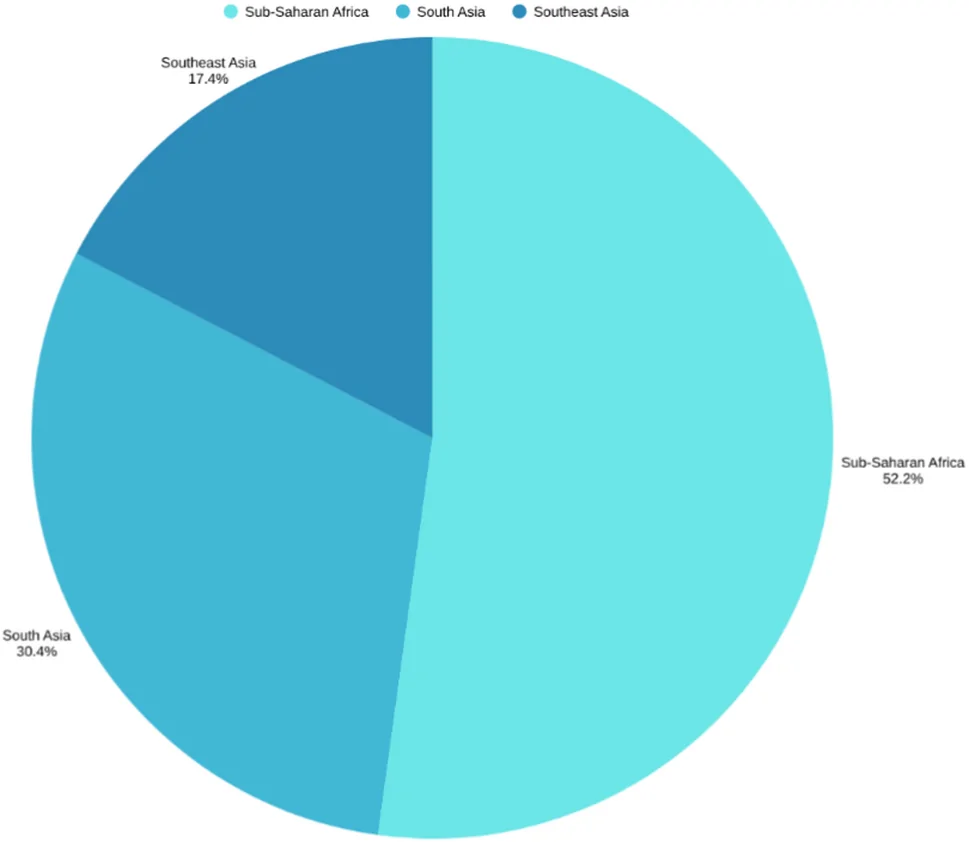
Distribution of papers by region

The primary studies were geographically distributed across three target regions: Sub-Saharan Africa, South Asia, and Southeast Asia (Fig. 2). Sub-Saharan Africa contributed 12 empirical studies (52.17%) conducted across Northern Ethiopia, Tanzania and Zanzibar, South Sudan, Ghana, The Gambia, Malawi, Nigeria, and Zimbabwe. Primary research in this region focused heavily on school WASH infrastructure deficits, cultural beliefs, and structural barriers to product access. South Asia accounted for 7 studies (30.44%), spanning India, Nepal, and Bangladesh. Key themes in this cluster centered on school preparedness for menstruation, government-led sanitary napkin distribution programs, and sociocultural taboos. In Southeast Asia, 4 primary studies (17.39%) examined context-specific challenges in Indonesia and Malaysia. Across all three regions, the included primary studies comprised a mix of qualitative, quantitative descriptive, non-randomised, and mixed-methods research designs.

### Risk of bias in studies

**Fig. 3:**
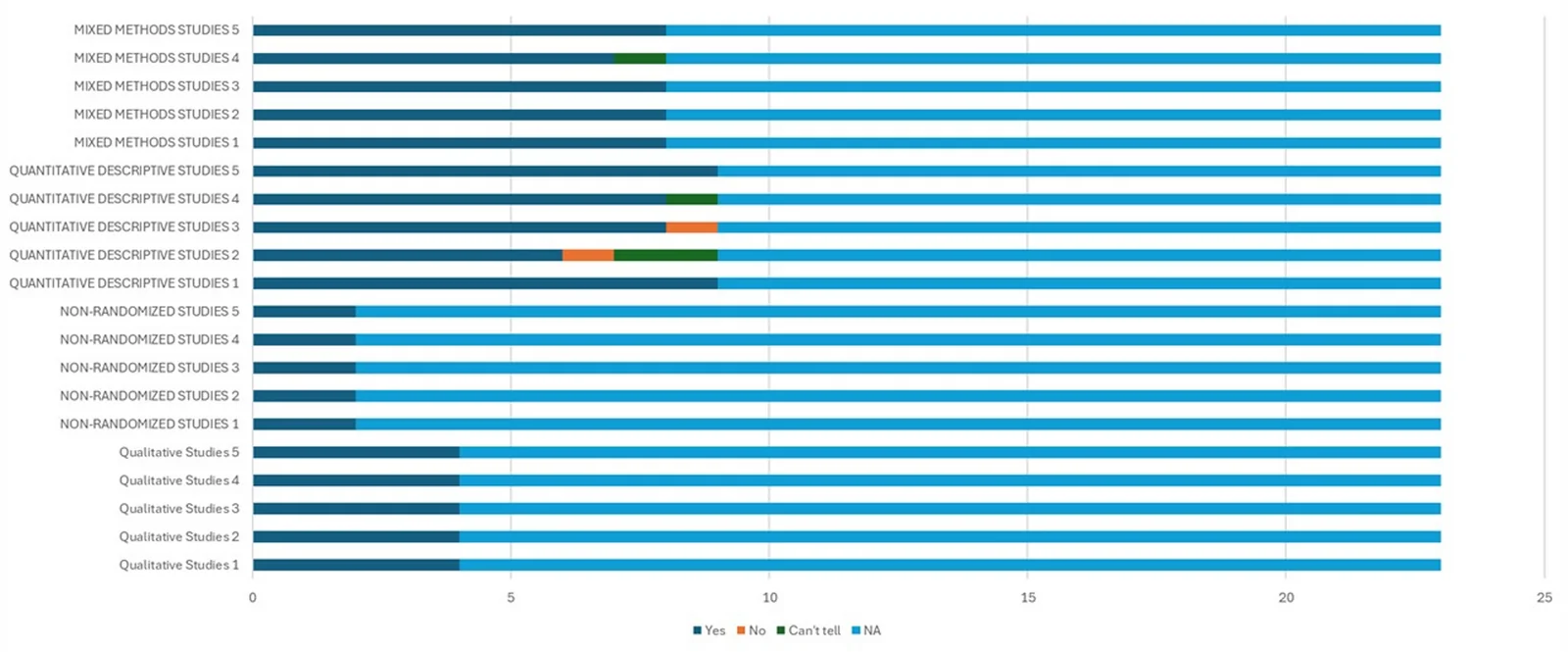
Results of the quality assessment of primary studies using the Mixed Methods Appraisal Tool (MMAT)

Methodological quality and risk of bias were systematically evaluated for all 23 included primary studies using the Mixed Methods Appraisal Tool (MMAT) across four distinct methodological categories (Fig. 3). Overall, the included studies demonstrated a high level of methodological rigor, with key variations observed across specific design domains. The qualitative studies (*n* = 4) and non-randomised quantitative studies (*n* = 2) demonstrated appropriate data collection, clear qualitative thematic mapping, and adequate controls for confounding variables.

Among the quantitative descriptive studies (*n* = 9), sampling strategies and statistical analyses were robust across all papers. However, domain-specific limitations were identified: three studies exhibited a high (*n* = 1) or unclear (*n* = 2) risk regarding target population representativeness, one study lacked appropriate measurement tools, and one study had an unclear risk regarding non-response bias. The mixed-methods studies (*n* = 8) exhibited strong structural integration across design components. Minor limitations were restricted to a single study, where it was unclear whether divergences between qualitative and quantitative findings were adequately addressed.

### Synthesis of results: the social-ecological model

The findings of the included studies were synthesized using a thematic narrative approach, structured according to the five interrelated levels of the Social-Ecological Model (SEM). This multi-level framework facilitates an examination of how individual behaviors are conditioned by interpersonal, institutional, community, and policy-level determinants.

### Individual level

#### Knowledge and menstrual literacy

Menstrual literacy varied across the included studies, although misconceptions regarding reproductive anatomy and menstrual biology remained common. Even where girls recognised menstruation as a natural biological process, important conceptual gaps persisted. Across several settings, relatively few participants correctly identified the uterus as the source of menstrual blood, indicating that basic biological understanding remained incomplete despite generally adequate awareness of menstruation [48–51].

Knowledge deficits were more pronounced in low-resource and humanitarian settings, where limited pre-menarche awareness left many girls unprepared for their first menstrual experience [52–55]. Across the included studies, higher menstrual literacy was consistently associated with older age, higher educational attainment, urban residence, previous exposure to menstrual health education, and higher maternal education. Greater menstrual knowledge was also associated with safer menstrual hygiene practices [48, 55–58].

#### Emotional experiences

Fear, anxiety, embarrassment, and shame were consistently reported across diverse settings, particularly among girls who had received little or no information before menarche. Many girls described their first menstrual experience as frightening, confusing, and emotionally distressing, while adolescents living in humanitarian settings reported additional psychological challenges associated with displacement, poverty, and limited access to menstrual resources [53, 54, 58, 59].

Fear of menstrual leakage emerged as a recurrent concern across the included studies and was associated with reduced classroom participation, reluctance to use school sanitation facilities, and menstruation-related school absenteeism [49, 60–64]. Adverse mental health outcomes were also reported, with dysmenorrhoea associated with increased anxiety, depressive symptoms, and psychological distress, particularly in contexts where access to menstrual products, privacy, and adequate hygiene facilities was limited [50, 54, 65].

#### Menstrual hygiene practices

Menstrual hygiene practices varied substantially across socio-economic and geographic settings. Disposable sanitary pads were the most commonly reported menstrual product in several studies, whereas girls living in rural, low-income settings more frequently relied on reusable cloths or improvised materials because commercial products were either unaffordable or unavailable [48, 49, 53, 54, 56, 60, 62].

Access to adequate water, sanitation, and hygiene (WASH) facilities influenced girls’ ability to practise safe menstrual hygiene. Reliable water supplies, private toilets, and appropriate washing facilities supported regular changing of menstrual materials, whereas inadequate school WASH infrastructure often prevented girls from changing during school hours and was associated with prolonged product use [48, 60, 64, 66]. Privacy concerns also affected the management of reusable menstrual materials. Although girls were aware of the hygienic benefits of drying cloths in sunlight, many reported drying them indoors or in concealed locations because of concerns about privacy, stigma, or personal safety, particularly in humanitarian settings [49, 50, 53, 54, 67].

#### Menstrual symptoms and school participation

Dysmenorrhoea was one of the most frequently reported menstrual health concerns across the included studies and was consistently associated with disruptions to school attendance and daily activities. Moderate-to-severe menstrual pain, including abdominal cramps, back pain, and fatigue, was commonly reported and was associated with menstruation-related school absenteeism [49, 50, 65, 67].

School absenteeism was also associated with fear of menstrual leakage, limited access to clean menstrual materials, inadequate school sanitation facilities, and embarrassment related to managing menstruation in school. These barriers were particularly evident in resource-constrained settings, where shortages of menstrual products and inadequate WASH infrastructure further limited girls’ ability to manage menstruation during school hours [53, 54, 62, 64, 68, 69].

Overall, the included studies demonstrated that girls’ menstrual experiences at the individual level were shaped by the interplay of menstrual knowledge, emotional wellbeing, symptom burden, and access to appropriate menstrual resources. These findings provide the foundation for understanding how interpersonal, institutional, community, and structural factors further influence menstrual health outcomes.

### Interpersonal level

The interpersonal environment played a central role in shaping adolescent menstrual health by influencing menstrual knowledge, emotional wellbeing, hygiene management, and help-seeking behaviours. Across the included studies, family members particularly, mothers and other female relatives were the primary sources of menstrual information and practical support, although the timing and quality of communication varied considerably. Peer relationships also influenced girls’ menstrual experiences by providing emotional and practical support while, in some settings, reinforcing menstrual stigma and social exclusion.

#### Family communication and menstrual knowledge

Across the included studies, mothers consistently emerged as the primary and most trusted source of menstrual information. They commonly provided explanations about menstruation, guidance on hygiene practices, and reassurance following menarche, while older sisters, grandmothers, and aunts also contributed to menstrual knowledge and practical support [48, 49, 52, 58, 67, 69].

Despite their central role, menstrual discussions frequently occurred only after menarche rather than beforehand, leaving many girls inadequately prepared for their first menstrual experience [52–54, 59]. Higher maternal education and living with a mother or female guardian were associated with greater menstrual knowledge and preparedness among adolescent girls [52, 56, 57, 69]. However, family communication was not universal. Some girls reported receiving little or no guidance at menarche, while conflict, displacement, and household instability further limited parent–daughter communication in crisis-affected settings [54, 68].

#### Family support

Beyond providing information, mothers and other female relatives frequently supported girls by supplying menstrual products, demonstrating hygienic practices, and helping to normalise menstruation [48, 49]. They were also commonly the first point of contact for menstrual pain and other menstrual health concerns. Greater perceived family support was associated with lower levels of anxiety and depression among adolescent girls [65, 69].

On the other hand, several studies described family environments characterised by silence, restrictive social norms, and limited emotional support. In these contexts, menstruation was associated with restrictions on girls’ mobility and social interactions, while poverty, displacement, and household economic hardship limited families’ ability to meet menstrual health needs. Financial constraints also reduced access to menstrual products, leading some families to share reusable materials or prioritise other household necessities over menstrual supplies [53–55, 62].

#### Peer relationships and menstrual stigma

Peers represented an important source of information, reassurance, and practical support. Girls frequently relied on friends for advice, reminders about hygiene practices, and emergency menstrual supplies, and many reported feeling more comfortable discussing menstruation with classmates than with adult family members [48, 60, 68]. Positive peer relationships were also associated with improved menstrual hygiene practices and better psychological wellbeing [65].

Despite these supportive relationships, menstrual stigma often limited girls’ willingness to seek support from their peers. Fear of negative judgement encouraged many girls to conceal their menstrual status and avoid requesting emergency menstrual supplies [54, 60]. Several studies also reported teasing, bullying, and public humiliation by male peers, which were associated with increased anxiety, reduced classroom participation, and menstruation-related school absenteeism [53, 61–63]. Fear of being identified as menstruating also influenced everyday behaviours, including concealing menstrual products and avoiding routine activities that might reveal their menstrual status, such as carrying water to school sanitation facilities [66].

Collectively, the included studies demonstrate that interpersonal relationships influenced girls’ menstrual preparedness, confidence, hygiene management, emotional wellbeing, and school participation. Supportive family communication and positive peer relationships were associated with more favourable menstrual experiences, whereas limited communication, restrictive social norms, and menstrual stigma were associated with poorer menstrual health outcomes.

### Institutional Level

Institutional factors, particularly school environments and institutional preparedness, influenced adolescent menstrual health by shaping access to menstrual health education, water, sanitation, and hygiene (WASH) facilities, menstrual products, and supportive school environments. Across the included studies, schools served as an important setting for menstrual health promotion. However, deficiencies in WASH infrastructure, limited institutional support, inadequate teacher training, and inconsistent programme implementation frequently constrained girls’ ability to manage menstruation safely and with dignity.

#### School WASH infrastructure

Inadequate school WASH infrastructure was one of the most consistently reported barriers to effective menstrual hygiene management. Common challenges included unreliable water supplies, lack of soap, inadequate privacy, damaged sanitation facilities, absence of dedicated changing spaces, and limited options for the safe disposal of menstrual materials [49, 50, 52–54, 60, 66]. These deficiencies often prevented girls from changing menstrual materials during school hours, resulting in delayed changing, prolonged use of menstrual products, or returning home before managing menstruation [61, 64, 66]. Some girls also reported reducing their fluid intake to avoid using school sanitation facilities or leaving school early because of inadequate facilities [53, 66].

The management of used menstrual materials was further limited by inadequate disposal infrastructure. The absence of disposal bins, incinerators, or formal waste management systems led girls to flush used materials down toilets, discard them in pit latrines or open areas, or carry them home for disposal to avoid embarrassment [61, 64, 66].

#### Menstrual education and teacher support

Schools also served as an important platform for menstrual health education, although the delivery of education varied considerably across settings. Several studies reported that menstrual education was absent, inconsistently delivered, or dependent on individual teacher initiative rather than being systematically integrated into the curriculum [52, 57]. In some settings, limited teacher training and low awareness of national menstrual health guidance further constrained programme delivery [52].

Where structured school-based menstrual health education was implemented, girls generally demonstrated better menstrual knowledge, more positive perceptions of menstruation, and improved menstrual hygiene practices [48, 57]. Teacher attitudes also influenced girls’ experiences. Male teachers were frequently described as reluctant to discuss menstruation because of embarrassment or prevailing cultural norms, whereas female teachers were commonly identified as trusted sources of practical guidance, emotional reassurance, and emergency menstrual supplies [52, 53, 61].

#### School-based programmes and institutional preparedness

Several studies evaluated school-based menstrual health programmes and institutional readiness to support menstruating students. Multi-component interventions combining menstrual health education, provision of menstrual products, and improvements in school WASH facilities were associated with greater menstrual literacy, increased confidence in managing menstruation, and improved school participation [49, 55, 70].

Despite these positive findings, implementation was frequently constrained by inadequate resources, insufficient staff training, and unclear institutional responsibilities [52, 57]. These challenges were particularly evident in crisis-affected settings, where schools often lacked emergency menstrual supplies, private changing spaces, and appropriate psychosocial support for girls [53, 54].

The availability of menstrual products within schools also varied considerably. Although some schools-maintained emergency stocks of sanitary pads, routine distribution was often affected by financial constraints, inconsistent supply chains, and the absence of formal institutional policies [49, 60, 64, 66]. School staff generally recognised menstruation as a contributor to school absenteeism; however, institutional responses were largely reactive, and few schools had established standardised procedures or comprehensive support systems to address girls’ menstrual health needs [53, 61].

Collectively, the included studies demonstrate that institutional factors, including school WASH infrastructure, menstrual health education, teacher support, and programme implementation influenced girls’ ability to manage menstruation at school and participate fully in educational activities.

### Community level

Community-level factors influenced adolescent menstrual health by shaping social norms, cultural beliefs, gender expectations, and community attitudes towards menstruation. Across the included studies, menstrual stigma, restrictive cultural practices, and limited community awareness affected girls’ menstrual knowledge, hygiene practices, emotional wellbeing, and participation in daily activities.

#### Menstrual stigma and cultural norms

Across diverse settings, menstruation was commonly regarded as a private or taboo subject, limiting open discussion and contributing to misinformation. Girls frequently reported that menstruation was expected to remain hidden from boys, male family members, and the wider community, leading to embarrassment, reluctance to seek support, and delayed access to accurate menstrual information [54, 61, 63]. In several studies, menstruation was also associated with beliefs of ritual impurity or uncleanliness, which influenced girls’ social interactions and menstrual management practices [53, 54, 59, 69].

Community stigma also influenced menstrual hygiene behaviours. To avoid public visibility and social judgement, girls commonly concealed menstrual products while travelling to school, carefully wrapped used menstrual materials before disposal, and avoided drying reusable cloths outdoors despite recognising the hygienic benefits of sun drying [49, 58, 67]. Similar concealment practices were reported in resource-constrained environments, where inadequate sanitation infrastructure and limited privacy influenced girls’ menstrual management practices [54].

#### Menstrual restrictions and gender norms

Several studies described cultural and gender-based restrictions that limited girls’ participation in household, educational, social, and religious activities during menstruation. Restrictions commonly included exclusion from places of worship, participation in religious ceremonies, food preparation, and interactions with male family members [59, 69]. In some settings, the onset of menarche was associated with increased parental supervision, restricted mobility, and greater expectations regarding modesty, reflecting cultural perceptions of menstruation as a marker of transition to adulthood and marriage [53, 54].

#### Community awareness and male engagement

Several studies highlighted the influence of broader community awareness on adolescent menstrual health. Limited menstrual knowledge among men and boys contributed to stigma, teasing, and discriminatory behaviours, and many girls reported discomfort discussing menstruation with fathers, brothers, and male teachers because menstruation was widely perceived as a female-only issue [61, 63].

Conversely, studies evaluating community and school-based awareness initiatives reported that educating boys about menstruation was associated with reduced teasing, greater empathy, and more supportive school environments [52, 63]. Community sensitisation programmes involving parents, religious leaders, and local organisations also reported improvements in menstrual awareness and reductions in menstrual misconceptions [61, 70].

Collectively, the included studies demonstrate that community-level factors, including menstrual stigma, cultural norms, gender expectations, and community awareness influenced girls’ menstrual experiences, hygiene practices, and social participation.

### Policy and structural level

Policy and structural factors influenced adolescent menstrual health through national policies, public investment in water, sanitation, and hygiene (WASH), access to menstrual products, and broader socio-economic conditions. Across the included studies, several countries had introduced policies and programmes to support menstrual health education, improve school WASH infrastructure, and increase access to menstrual products; however, implementation varied considerably across settings.

#### National policies and programme implementation

Several studies described national or regional policies aimed at strengthening menstrual health education, improving school WASH infrastructure, and supporting the provision of menstrual products [52, 70]. However, implementation was inconsistent across regions and schools, resulting in variable access to menstrual health services. Limited awareness of national menstrual health guidelines, inadequate dissemination of implementation materials, and differences in institutional capacity were reported as barriers to programme implementation [52, 57, 64].

Implementation challenges were further reported in settings experiencing humanitarian crises, where weakened health and education systems limited access to menstrual products, sanitation facilities, and institutional support despite the inclusion of menstrual health within humanitarian response efforts [53, 54].

#### Economic barriers and structural inequalities

The affordability of commercial menstrual products emerged as one of the most consistently reported structural barriers across the included studies. Girls from economically disadvantaged households frequently relied on reusable cloths or improvised materials because disposable menstrual products were unaffordable, while competing household priorities further limited regular access to menstrual products [54, 56, 62, 67, 68]. Although school-based and government-supported product distribution programmes reduced immediate financial barriers, their implementation was frequently affected by inconsistent product availability and irregular supply chains [49, 70].

Broader structural factors, including poverty, geographic isolation, educational disparities, and conflict- or displacement-related disruptions, were associated with inequalities in menstrual health resources and support. Girls living in rural communities, urban informal settlements, and settings affected by conflict and displacement commonly experienced limited access to functional sanitation facilities, menstrual health information, and affordable menstrual products [53, 54, 56]. Similarly, girls attending under-resourced schools reported poorer WASH infrastructure, fewer opportunities for menstrual health education, and less institutional support than those in better-resourced schools [57, 66].

Collectively, the included studies demonstrate that policy implementation, economic access to menstrual products, and broader structural conditions influenced girls’ access to menstrual health resources and support across diverse settings.

### Cross-level synthesis

The included studies demonstrated that adolescent menstrual health was influenced by interacting factors operating across all levels of the Social Ecological Model. Individual menstrual knowledge, emotional wellbeing, and hygiene practices were closely associated with interpersonal relationships, school environments, community norms, and broader policy and structural conditions.

Across the included studies, supportive family communication, positive peer relationships, and trusted teachers were associated with greater menstrual preparedness, increased confidence, and improved menstrual hygiene practices. In contrast, limited communication, peer teasing, menstrual stigma, and inadequate emotional support were associated with anxiety, negative menstrual experiences, and reduced participation in school activities [48, 53, 63]. Similarly, girls’ ability to manage menstruation safely at school was influenced by institutional factors, including access to private sanitation facilities, reliable water supplies, menstrual products, and supportive school environments [49, 60, 66].

Community-level influences, including menstrual stigma, restrictive gender norms, and limited community awareness, further shaped girls’ menstrual experiences by affecting communication, help-seeking behaviours, and menstrual management practices [54, 59, 61]. At the policy and structural level, disparities in policy implementation, economic access to menstrual products, WASH infrastructure, and broader socio-economic conditions were associated with differences in girls’ access to menstrual health information, resources, and institutional support [52, 53, 70].

Collectively, the included studies demonstrate that menstrual health outcomes were shaped by the interaction of individual, interpersonal, institutional, community, and policy-level influences, with factors operating across multiple levels of the Social Ecological Model.

## Discussion

Across the 23 studies from South Asia, Sub-Saharan Africa, and Southeast Asia, evidence shows that menstrual knowledge has improved; however, translating this knowledge into confident, hygienic practice remains dependent on enabling social and structural conditions. Many girls recognise menstruation as physiological, but understanding is shallow where pre-menarche education is rare [16, 48, 60, 71]. Although greater menstrual knowledge was generally associated with improved menstrual hygiene practices, the findings indicate that knowledge alone was insufficient where girls lacked affordable menstrual products, private sanitation facilities, and reliable access to water and safe disposal mechanisms [67–69, 72, 73].

At the interpersonal level, mothers remain the primary educators, with minimal father and daughter dialogue and uneven teacher or peer involvement [16, 69, 74]. Supportive peers and trained teachers improve comfort, while teasing and restricted toilet access undermine dignity [63, 75, 76]. Low family or peer support correlates with anxiety and depression, highlighting reassurance as a protective factor [65].

Organisational deficits form the main bottleneck. The poor WASH conditions, including the absence of locks, water, soap, privacy, and disposal facilities, limit safe management, prompting absenteeism and insecurity [44, 49, 77]. Furthermore, standard measurement approaches often fail to fully capture the true burden of menstruation-related school absenteeism in low-income settings [78]. Many schools meet “improved sanitation” criteria but lack usability and maintenance [50, 63]. In humanitarian contexts, unsafe, unlit facilities turn menstruation into a dignity crisis [54].

Community stigma remains pervasive: menstruation is often seen as polluting, restricting participation and reinforcing secrecy [49, 52, 53, 65]. Caste, religion, and poverty determine access to products and water, thereby perpetuating inequity [16, 79]. Advocacy has raised visibility, but engagement of men and faith leaders is limited [76, 80]. At the policy level, intent outpaces implementation. While many countries incorporate MHM into their WASH strategies, financing, monitoring, and teacher training remain inconsistent and donor-driven [48, 60, 75, 76, 80, 81]. Programmes often prioritise hardware over sustained operations and maintenance (O&M), stigma reduction, and mental health support [49, 61, 70].

Collectively, weak policy and infrastructure reinforce interpersonal silence and community stigma, constraining the knowledge-to-practice pathway. Effective progress occurs when “hardware plus software” aligns private, reliable WASH, trained, inclusive teachers, steady product supply, and stigma reduction within system budgets [38, 54, 80]. Menstrual health thus requires coordinated, multi-level action that integrates rights-based policies, gender-sensitive facilities, social support, and evaluation of dignity and participation beyond mere attendance [48, 81, 82].

### Limitations

This review was limited to studies published in English and relied mainly on self-reported data, which may introduce bias. Variation in study designs and outcomes restricted the meta-analysis and cross-country comparison. Additionally, while terms like ‘menstrual health’ were included, the explicit keyword ‘Menstrual Health and Hygiene (MHH)’ was not utilized in the initial search string, which may have excluded a small subset of recently published literature using this updated nomenclature. Nonetheless, the review provides an integrative understanding of how school-based WASH and social environments influence menstrual health among adolescent girls in low- and middle-income settings.

## Conclusion

This review suggests that menstrual health and hygiene among adolescent girls were influenced by interdependent ecological factors, rather than individual knowledge alone. While awareness and attitudes have improved across various settings, deficits in infrastructure, social support, and policy implementation continue to hinder the safe and dignified management of these issues. Knowledge often remains superficial and moderated by affordability, fear, and stigma; interpersonal silences, particularly among fathers and male teachers, limit open dialogue; and inadequate school WASH, privacy, and disposal facilities hinder behavioural translation.

Community stigma and restrictive norms perpetuate secrecy, while policy frameworks remain fragmented, underfunded, and poorly monitored. These interconnected barriers create a reinforcing cycle where progress at one level is undermined by neglect at another. Sustainable improvement requires coordinated, multi-level action rights-based policies with dedicated financing and accountability, gender-sensitive and well-maintained school environments, trained educators, and active engagement of families and community leaders. Future research should also examine cost-effectiveness, disability inclusion, and long-term psychosocial outcomes to strengthen evidence-informed policy and programming.

## Supplementary Information

Supplementary Material 1.

Supplementary Material 2.

Supplementary Material 3.

Supplementary Material 4.

## Data Availability

All data accessed during this study are included in this article and its supplementary information files.

## Abbreviations

LMICs: Low- and Middle-Income Countries
MHM: Menstrual Hygiene Management
NGO: Non-Governmental Organization
O&M: Operations and Maintenance
SDG: Sustainable Development Goal
SEM: Socio-Ecological Model
UNICEF: United Nations Children’s Fund
WASH: Water, Sanitation and Hygiene
WHO: World Health Organization
