# Supplementary material for: School-based WASH facilities and menstrual hygiene management: a systematic review of impacts on adolescent girls in South Asia, Sub-Saharan Africa, and Southeast Asia
Source: BMC Womens Health. 2026 Aug 17;26:608. doi: 10.1186/s12905-026-04796-2 (PMC13757013; doi:10.1186/s12905-026-04796-2)
Supplement: Supplementary file 1 [file 12905_2026_4796_MOESM1_ESM.pdf]

Detailed Search Strategies by Database

PubMed

("Menstrual Hygiene Management"[All Fields]

OR "Menstrual Health"[All Fields]

OR "Menstruation"[MeSH Terms]

OR "Menstruation"[All Fields])

AND

("Schools"[MeSH Terms]

OR school*[All Fields]

OR education[All Fields]

OR "school attendance"[All Fields]

OR adolescent*[All Fields])

AND

("WASH"[All Fields]

OR sanitation[All Fields]

OR hygiene[All Fields]

OR "school sanitation"[All Fields]

OR toilet*[All Fields]

OR water[All Fields])

AND

("South Asia"[All Fields]

OR "Sub-Saharan Africa"[All Fields]

OR "Southeast Asia"[All Fields])

Scopus

TITLE-ABS-KEY(

("menstrual hygiene management"

OR "menstrual health"

OR menstruation)

AND

(school*

OR education

OR "school attendance"

OR adolescent*)

AND

(WASH

OR sanitation

OR hygiene

OR "school sanitation"

OR toilet*

OR water)

AND

("South Asia"

OR "Sub-Saharan Africa"

OR "Southeast Asia")

)

Web of Science

TS=(

("menstrual hygiene management"

OR "menstrual health"

OR menstruation)

AND

(school*

OR education

OR "school attendance"

OR adolescent*)

AND

(WASH

OR sanitation

OR hygiene

OR "school sanitation"

OR toilet*

OR water)

AND

("South Asia"

OR "Sub-Saharan Africa"

OR "Southeast Asia")

)

JSTOR

(("menstrual hygiene management"

OR "menstrual health"

OR menstruation)

AND

(school*

OR education

OR "school attendance"

OR adolescent*)

AND

(WASH

OR sanitation

OR hygiene

OR "school sanitation"

OR toilet*

OR water)

AND

("South Asia"

OR "Sub-Saharan Africa"

OR "Southeast Asia"))

Search Limits Applied

Across all databases, searches were limited to:

English-language publications

Studies published between January 2009 and October 2025

Grey Literature Sources

Additional grey literature searches were conducted using:

Google Scholar

World Health Organization (WHO) reports

UNICEF publications

UNESCO reports

Governmental and non-governmental organisation (NGO) reports related to menstrual health and school-based WASH programmes
